# Occurring of *In Vitro* Functional Vasculogenic Pericytes from Human Circulating Early Endothelial Precursor Cell Culture

**DOI:** 10.1155/2015/943671

**Published:** 2015-05-06

**Authors:** Silvia Cantoni, Francesca Bianchi, Margherita Galletti, Elena Olivi, Francesco Alviano, Nazzareno Galiè, Carlo Ventura

**Affiliations:** ^1^National Institute of Biostructures and Biosystems (NIBB), University of Bologna, 40138 Bologna, Italy; ^2^Department of Experimental, Diagnostic and Specialty Medicine (DIMES), University of Bologna, 40138 Bologna, Italy; ^3^Stem Wave Institute for Tissue Healing (SWITH), Gruppo Villa Maria (GVM) and Ettore Sansavini Health Foundation-ONLUS, Lugo, 48022 Ravenna, Italy

## Abstract

Pericytes are periendothelial cells of the microcirculation which contribute to tissue homeostasis and hemostasis by regulating microvascular morphogenesis and stability. Because of their multipotential *ex vivo* differentiation capabilities, pericytes are becoming very interesting in regenerative medicine field. Several studies address this issue by attempting to isolate pericyte/mesenchymal-like cells from peripheral blood; however the origin of these cells and their culture conditions are still debated. Here we showed that early Endothelial Progenitor Cells (EPCs) expressing CD45+/CD146+/CD31+ can be a source of cells with pericyte/mesenchymal phenotype and function, identified as human Progenitor Perivascular Cells (hPPCs). We provided evidence that hPPCs have an immunophenotype consistent with Mesenchymal Stem Cells (MSCs) from human adipose tissue (hASCs) and fetal membranes of term placenta (FM-hMSCs). In addition, hPPCs can be subcultured and exhibit expression of pluripotent genes (*OCT-4, KLF-4*, and *NANOG*) as well as a remarkable vasculogenic potential. Our findings could be helpful to develop innovative cell-based therapies for future clinical applications with distinct therapeutic purposes.

## 1. Introduction

Pericytes are becoming more and more interesting in regenerative medicine because of their multipotential* ex vivo* differentiation capabilities, in particular towards a vasculogenic lineage [1–4]. This population of resident vascular cells is embedded within the basement membrane surrounding the endothelial cells of the microvasculature, from precapillary arterioles to small collecting venules [5]. Pericytes provide plenty of functions to maintain tissue homeostasis and hemostasis, including capillary blood flow regulation, clearance of cellular debris, and regulation of vessel permeability. Noteworthy, pericytes stabilize and monitor the maturation of endothelial cells both by direct communication between the cell membrane and by paracrine signaling [1, 6].

Several studies address this issue by attempting to isolate pericyte/mesenchymal-like cells from peripheral blood [7–14]; however the origin of these cells and their culture conditions are still debated.

Asahara et al. in 1997 first described Endothelial Progenitor Cells (EPCs) in Peripheral Blood Mononuclear Cells (PBMCs) [15]. Two groups of EPCs have been defined in* in vitro* models: the “early outgrowth EPCs,” which are derived from the monocytes and express CD45 and CD14 on their surface, other than CD31, displaying an overlap between endothelial, monocyte, and macrophage cell markers; the “late outgrowth EPCs,” which are believed to be a subset of CD14−/CD34−/KDR− (Kinase insert Domain Receptor) cells that do not express CD45 and CD14 [16].

These two populations showed different characteristics and brought different contribution to neovasculogenesis. In particular early outgrowth EPCs are derived from the monocytic cell lineage and form clusters from which cells with a spindle-shaped morphology originate after as little as 7 days of culture. They possess transient proliferative potential* in vitro* and cannot be passaged [17–19].

Here we showed that this cell population can be a source of cells with pericyte/mesenchymal phenotype and function that we identified as human Progenitor Perivascular Cells (hPPCs). We provided evidence that these pericyte/mesenchymal-like cells have an immunophenotype consistent with Mesenchymal Stem Cells (MSCs) from different sources including human adipose tissue and term placenta; they can be subcultured and exhibit gene expression of pluripotency as well as a remarkable vasculogenic potential.

## 2. Methods

### 2.1. Isolation of PBMCs

According to the policies approved by the institutional review boards for human studies of local ethical committees, all blood samples (*n* = 10) from healthy adult donors were obtained after informed consent. Blood was drawn from a peripheral venipuncture into vacutainer tubes (BD, Biosciences, San Jose, CA, USA) containing ethylenediaminetetraacetic acid. PBMCs were isolated starting from 20 mL of whole blood with Lympholyte-H (CEDARLANE Laboratories, Ontario, Canada) following the manufacturer's instructions. Briefly, blood was diluted 1 : 2 with Phosphate Buffered Saline (PBS) and stratified onto the equal volume of Lympholyte. After centrifugation, the ring containing PBMCs was collected and transferred into a new tube. Whole blood hemolysis with isotonic ammonium chloride solution (0.15 M ammonium chloride; 10 mM potassium bicarbonate; 0.1 mM ethylenediaminetetraacetic acid) was performed to eliminate erythrocytes. Totally four washes were performed before counting and seeding PBMCs.

### 2.2. Isolation of Pericyte/MSC-Like Cells from PBMCs

Cell counts were performed with Millipore's Scepter automated handheld cell counter (Millipore, Billerica, MA, USA), which allowed us to distinguish almost two different cell population based upon diameter dimension.

PBMCs containing 6-7 × 10^6^ cells with diameter greater than 8.6 *μ*m were seeded into 12-well plate precoated with 2.5 *μ*g/mL fibronectin (Sigma, Indianapolis, IN, USA), in 2 mL of MicroVascular Endothelial Cell Growth Medium-2 (EGM-2-MV) (Lonza, Basel, Switzerland) to obtain early EPCs, as previously described [20, 21].

After 7 days the medium was replaced with Minimum Essential Medium with *α*-modification (*α*-MEM) supplemented with 20% heat-inactivated Fetal Bovine Serum (FBS), antibiotics (100 units/mL penicillin, 100 units/mL streptomycin), and L-glutamine (2 mM) and incubated at 37°C in a humidified atmosphere with 5% CO_2_ (all reagents from Lonza).

The medium was changed after 7 days and subsequently every three days until cells reach confluence, needing to be trypsinized for subculturing or cryopreservation.

### 2.3. Flow Cytometry

For flow cytometry analysis, cells were incubated with 1 *μ*g/10^6^ cells of fluorescent antibodies for 40 min at 4°C in the dark. The antibodies used for flow cytometry were anti-CD31, anti-CD90, anti-CD105, anti-CD133, anti-CD146, anti-PDGFRbeta (all from BioLegend, San Diego, CA, USA), anti-CD14, anti-CD29, anti-CD34, anti-CD44, anti-CD45, anti-CD73, anti-CD166, anti-CD271 (all from BD Biosciences), and anti-NG2 (R&D Systems). After washing, cells were analyzed on a flow cytometer (FACSAria, BD Biosciences) by collecting 10,000 events, and the data were analyzed using the FACSDiva Software (BD Biosciences).

For the analysis of whole blood, 100 *μ*L was used for each test: after staining with appropriate antibodies combination, cells were subjected to hemolysis to eliminate erythrocytes and washed before the acquisition.

Sorting strategy was used to isolate CD146+ and CD146− cells. Cells were stained with anti-CD146 antibody, as described above. Stained cells were washed once with 1% Bovine Serum Albumin (Sigma-Aldrich) in PBS and sorted immediately on a fluorescence-activated cell sorter FACSAria using FACSDiva software.

A 100 *μ*m ceramic nozzle, sheath pressure of 20–25 PSI (Pounds per Square Inch), and an acquisition rate of 300–500 events per second were used as conditions. Prior to sorting, the nozzle, sheath, and sample lines were sterilized with 70% ethanol, followed by washes with sterile PBS to remove remaining decontaminant. FSC-peak (height) versus FSC-integral (area) gating was applied to exclude doublets for cell sorting. Sorted cells were immediately used for vasculogenesis assay.

### 2.4. Isolation and Culture of Fetal Membrane-Derived Human MSCs (FMhMSCs)

FMhMSCs have been isolated as previously described [22]. Briefly, term placenta obtained from caesarian sections was rapidly rinsed in PBS containing penicillin and streptomycin and used immediately. Pieces from fetal membranes were minced and digested for 10 min in *α*-MEM with 0.25% trypsin-EDTA, 10 U/mL DNase I, and 0.1% collagenase (all from Sigma-Aldrich). Tissues were pipetted vigorously up and down avoiding foam for 5 min; larger pieces of tissue were allowed to settle under gravity for 5 min. Each supernatant was transferred to a fresh tube, neutralized with FBS, and then spun at 1,000 ×g for 10 min. Each pellet was resuspended in 5 mL of *α*-MEM containing 20% FBS, 100 units/mL penicillin, 100 units/mL streptomycin, and 2 mM L-glutamine. FMhMSCs were seeded into culture flasks and grown at 37°C in 5% CO_2_. Nonadherent cells were removed after 1 week and medium (with 10% FBS) was changed subsequently every 4 days.

### 2.5. Isolation and Culture of Human Adipose Tissue-Derived Stem Cells (hASCs)

According to the policy approved by the local ethical committee, all tissue samples were obtained after informed consent. Human subcutaneous adipose tissue samples were obtained from lipoaspiration procedures. After washing, lipoaspirates were digested with 0.2% collagenase A type I solution (Sigma-Aldrich), under gentle agitation for 45 min at 37°C, and centrifuged at 800 ×g for 10 min to separate the Stromal Vascular Fraction (SVF) from adipocytes. If necessary, the SVF was treated with red blood cell lysis buffer for 5 min at 37°C and then centrifuged again. The supernatant was discarded and the cell pellet was resuspended and seeded in culture flasks in *α*-MEM supplemented with 10% heat-inactivated FBS, 100 units/mL penicillin, 100 units/mL streptomycin, and 2 mM L-glutamine and incubated at 37°C in a humidified atmosphere with 5% CO_2_. When the cultures were near confluence, the cells were detached by treatment with trypsin-EDTA 1X (Sigma-Aldrich), characterized, subcultured, and used at passage numbers 3–5.

### 2.6. Gene Expression Analysis

Total RNA was extracted using RNeasy Microkit (Qiagen, Milan, Italy), and 1 *μ*g was reverse-transcribed into cDNA in a 21 *μ*L reaction volume with SuperScriptTM III Reverse Transcriptase (Life Technologies, Carlsbad, CA, USA). To assess gene expression, 2 *μ*L of cDNA was used for real-time PCR performed with the SYBR Green I FastStart kit (Lightcycler FastStart DNA MasterPLUS SYBR Green I; Roche Diagnostics, Mannheim, Germany) using a LightCycler system (Roche Diagnostics), following the manufacturer's instructions.

Primers (0.25 *μ*M) used were as follows:* GAPDH* forward 5′-CAGCCTCAAGATCATCAGCA-3′ and reverse 5′-TGTGGTCATGAGTCCTTCCA-3′;* KDR* forward 5′-CTGCAAATTTGGAAACCTGTC-3′ and reverse 5′-GAGCTCTGGCTACTGGTGATG-3′;* KLF-4* forward 5′-GCCCAATTACCCATCCTTCCT-3′ and reverse 5′-CGATCGTCTTCCCCTCTTTG-3′;* NANOG* forward 5′-CCTTCCTCCATGGATCTGCTT-3′ and reverse 5′-CTTGACCGGGACCTTGTCTTC-3′;* OCT-4* forward 5′-CAATTTGCCAAGCTCCTGAAG-3′ and reverse 5′-AAAGCGGCAGATGGTCGTT-3′; primers for* MMP-2* and* NOS3* were purchased from Qiagen (human QuantiTect Primer Assay).

Obtained Ct values were normalized using* glyceraldehyde-3-phosphate dehydrogenase* (*GAPDH*) as an index of cDNA content after reverse transcription. Samples were run in duplicate, and the average threshold cycle (Ct) value was used for calculations.

### 2.7. *In Vitro* Vasculogenesis

Analysis of capillary-like tube formation was performed using semisolid medium Matrigel (BD Biosciences) as previously described [23]. Cells were seeded into 96-well plates precoated with 50 *μ*L of Matrigel at a density of 30,000 cells/cm^2^ in EGM-2 medium (Lonza) to induce vasculogenesis.

To investigate whether hPPCs might support capillary-like tube formation somehow interacting with endothelial cells, we set up a vasculogenesis assay by coculturing hPPCs and HUVECs (Human Umbilical Vein Endothelial Cells, Lonza). Before coculture, hPPCs and HUVECs were labeled, respectively, with vital dyes PKH2 Green and PKH26 Red Fluorescent Cell Linker Kits (Sigma-Aldrich), following the manufacturer's instructions. After the staining procedure, the cells were mixed in a 1 : 3 ratio (hPPCs : HUVECs) and 10,000 total cells (30,000 cells/cm^2^) were seeded for each well in a total volume of 150 *μ*L of EGM-2/*α*-MEM containing 2% FBS (1 : 1, vol : vol).

Capillary-like structures were observed at regular time intervals, starting from 3 hours till 7 days, and photographed using an inverted optical microscope (Eclipse TS100, Nikon, Japan) equipped with a digital sight camera (NIS-Elements D 3.0 software, Nikon) and fluorescence filters.

### 2.8. Immunofluorescence

Seven days after culturing in semisolid medium, the tubule-like structures were recovered from Matrigel by using Cell Recovery Solution (BD Biosciences), at 4°C for 1 hour following the manufacturer's instructions. Then, they were fixed for fluorescence immunostaining with ice-cold acetone : methanol (7 : 3) for 10 min. Nonspecific antibody binding sites were blocked by incubating cells with 4% BSA/PBS for 1 hour at room temperature. The blocking solution was then carefully removed and cells were incubated for 45 min at 37°C with monoclonal mouse anti-human CD34 conjugated to FITC (1 : 25 dilution, BD Biosciences) or with monoclonal rabbit anti-human VEGF Receptor 2 (VEGFR2 or KDR, 1 : 400 dilution, Cell Signaling). Following several washing in PBS/Tween 20 (0.25%), the latter samples were incubated with goat anti-rabbit IgG Alexa Fluor 488 (1 : 1,000 dilution, Invitrogen) for 45 min at 37°C in the dark. After this incubation time, cells were extensively washed, and then the nuclei were counterstained with DAPI solution (0.1 *μ*g/mL final concentration, Invitrogen). The samples were mounted with antifade reagent (ProLong Gold, Invitrogen). CD34-negative cells were used as control for CD34 staining, while VEGFR2 negative control was done by omitting the primary antibody. Samples were observed under a fluorescence microscope (Nikon) and images acquired and merged with a digital camera through the imaging software NIS-Elements.

### 2.9. Data Analysis

Relative quantification of mRNA expression has been calculated with the comparative Ct method using the “ΔΔCt method” for comparing relative expression results between the different groups in real-time PCR [24]. Significant differences among various groups were determined by ANOVA followed by Bonferroni post-hoc test. Values were expressed as mean ± standard error. Differences at *P* < 0.05 and *P* < 0.01 were considered to be statistically significant and extremely significant, respectively.

## 3. Results

### 3.1. Isolation and Characterization of Pericytes/Mesenchymal Progenitors from Peripheral Blood

Freshly isolated PBMCs obtained from 20 mL of healthy donors were composed mainly by small- and large-size cell populations, and the total number ranged from 11.7 to 17.6 million with diameter from 5 to 15.6 *μ*m. Since hemolysis eliminated erythrocytes, we ascribed the presence of small diameter cells to platelet contamination, so we seeded those cells with a diameter greater than 8.5 *μ*m, whose count was 6-7 million.

**Figure 1:**
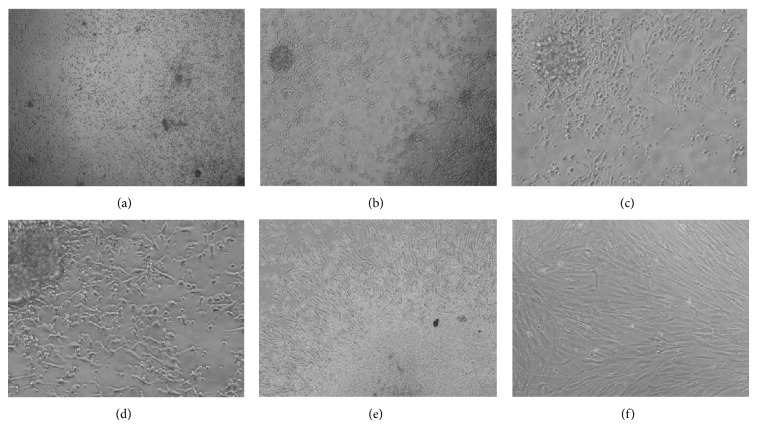
Photomicrograph of cells obtained from the early Endothelial Progenitor Cells (EPCs). (a) Mononuclear cells 24 hours after seeding into fibronectin coated plastic surface (40x magnification). (b)–(d) CFUs-Hill or early outgrowth EPCs after 7 days of culture. (b) 100x magnification; (c) 200x and (d) 400x magnifications. (e) CFUs-Hill and human Progenitor Perivascular Cells (hPPCs) progeny after 10 days of culture (40x magnification). (f) Pericyte/MSC-like cells, hPPCs, at the first passage (100x magnification).

Cells cultured in EGM-2-MV were aggregated to form sphere-like structures within the first days, and some of these were attached to fibronectin coated wells (Figure 1(a)).

After 7 days of culture the attached sphere-like structures showed a central cluster of rounded and flat cells with a radial progeny of spindle-shaped cells (Figures 1(b)–1(d)), and they resembled Colony Forming Units (CFUs) described previously by Hill et al. [25]. On day 7 the *α*-MEM supplemented with 20% FBS was used for culturing cells, and starting from day 10 an adherent spindle-shaped cell population became predominant (Figure 1(e)). Even after thawing, cells showed proliferation with 36–48 hours of population doubling (Figure 1(f)). Cells were expanded and cryopreserved until passage 5 without any visible morphological alterations.

### 3.2. Immunophenotypic Profile

The cytometric analysis, carried out in whole blood after depleting erythrocytes with hemolysis, did not reveal any distinct population with pericyte/MSC profile showing that CD146+/CD31−/CD34−/CD45− cells were less than 0.05%. We obtained the same result when we analyzed PBMCs just isolated and not seeded, where the CD146+/NG2+/PDGFRbeta+ cells were 0.1% ± 0.04.

**Figure 2:**
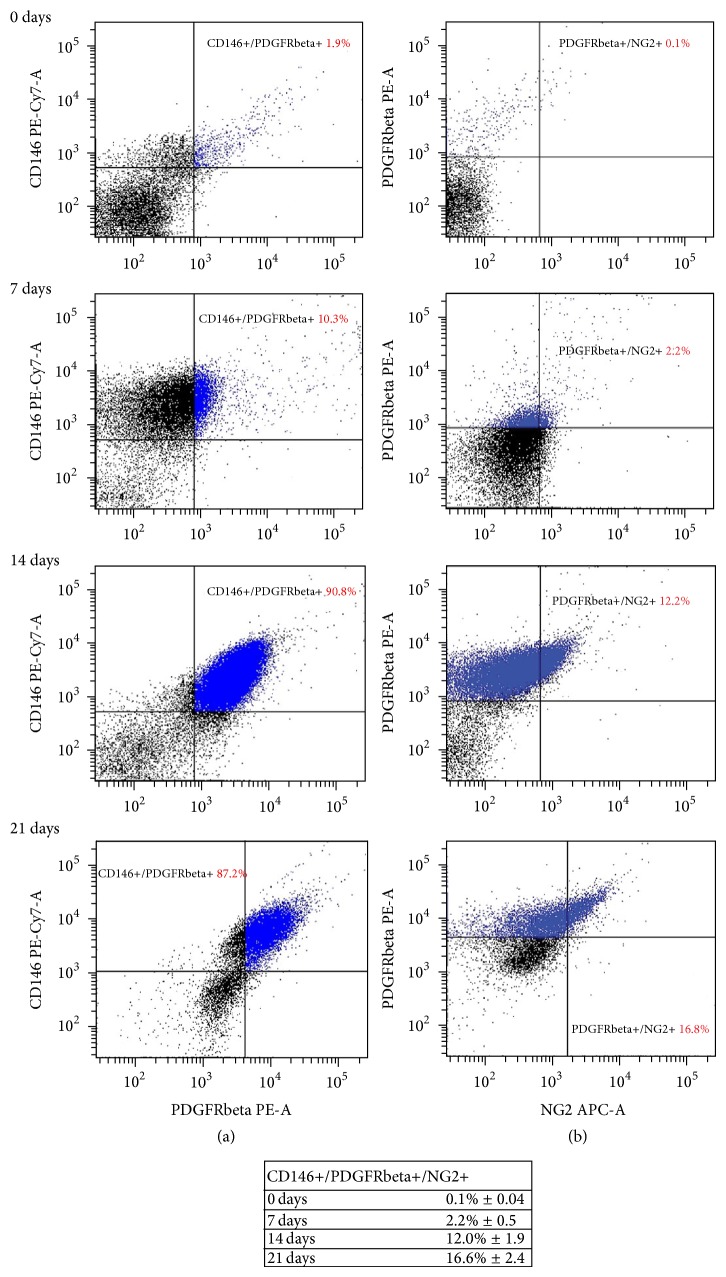
Time-course cytofluorimetric analysis of pericytic profile (CD146+/PDGFRbeta+/NG2+). Dot plot of CD146/PDGFRbeta (a) and PDGFRbeta/NG2 (b) coexpression in cells at different time points. Cells were analysed at time zero (PBMCs, just isolated not seeded), and after seeding (CFUs-Hill/early EPCs) at 7, 14, and 21 days. The dot plots are representative of 4 independent experiments. The table displays the total of cells with CD146+/PDGFRbeta+/NG2+ pattern at different times as indicated.

On day 7, the heterogeneous population resulting from CFUs-Hill showed the presence of interesting elements expressing the following profiles: CD146+/CD31+/CD45+/CD34− (70.5% ± 8.7), CD146+/CD31−/CD45+/CD34− (3.1% ± 0.7), and CD146+/NG2+/PDGFRbeta+ (2.2% ± 0.5). Interestingly, the percentage of this last population, showing an immunophenotype typical of pericytes, increased throughout the* in vitro* culture, reaching the 12% ± 1.9 after 14 days and the 16.6 ± 2.4 after 21 days (Figure 2).

While nonadherent cells showed principally a hematopoietic profile (data not shown) the adherent spindle-shaped cells, pericyte/MSC-like cells that we identified as human Progenitor Perivascular Cells (hPPCs), were positive when analyzed at the first passage for PDGFRbeta (42.8% ± 7.7), CD146 (78% ± 8.7), CD105 (99.4% ± 0.4), CD73 (99.3% ± 0.6), CD29 (99.9% ± 0.1), CD44 (99.5% ± 0.3), CD166 (87.2% ± 1.7), CD90 (61.6% ± 8.5), and CD166 (87.2% ± 2.1) and negative for CD271 (2.4% ± 0.4), CD133 (0.4% ± 0.2) and the hematopoietic markers CD14 (1.1% ± 0.9), CD34 (2.9% ± 1.5), and CD45 (2.6% ± 1.1).

Even the immunophenotypic profile of subcultured hPPCs was consistent with those profiles proper to hASCs and FM-hMSCs (Table 1) and that reported in the literature for bone marrow-derived hMSCs [26].

### 3.3. Gene Expression Analysis

**Figure 3:**
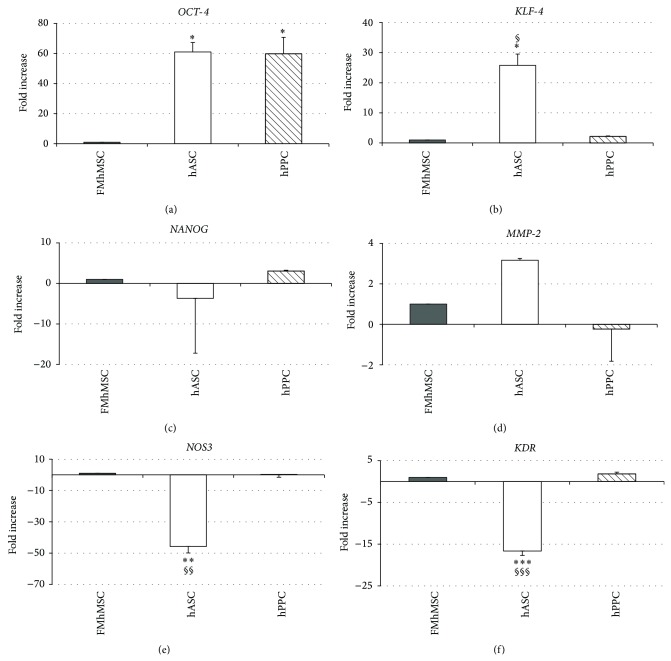
Comparative analyses of gene expression pattern of stemness and vasculogenic genes in hMSCs derived from placenta (FMhMSCs), adipose tissue (hASCs), and human Progenitor Perivascular Cells (hPPCs). Real-time PCR data of (a)* octamer-binding transcription factor 4* (*OCT-4*); (b)* Kruppel-like factor 4* (*KLF-4*); (c)* NANOG*; (d)* Matrix Metalloproteinase-2* (*MMP-2*); (e)* nitric oxide synthase 3* (*NOS3*); (f)* Kinase insert Domain Receptor* (*VEGF Receptor 2*) (*KDR*) are presented. Data were normalized to* GAPDH* housekeeping gene and expressed as relative fold change compared to FMhMSCs. Statistical analysis of four independent experiments was performed using ANOVA with Bonferroni post-hoc test: ^∗^ *P* < 0.05, ^∗∗^ *P* < 0.01 versus FMhMSCs; ^§^ *P* < 0.05, ^§§^ *P* < 0.01 versus hPPCs.

The immaturity/stemness of pericyte/MSC-like cells has been assessed by measuring the gene expression of* octamer-binding transcription factor 4* (*OCT-4*),* Kruppel-like factor 4* (*KLF-4*), and* NANOG* (Figure 3) specific markers used for characterizing embryonic and induced pluripotent stem cells [27, 28].

While hPPCs showed an increased* OCT-4* gene expression (Figure 3(a)),* KLF-4* (Figure 3(b)) and* NANOG* (Figure 3(c)) genes did not show any significant variation as compared to FMhMSCs.

Interestingly,* Matrix Metalloproteinase-2* (*MMP-2*),* nitric oxide synthase 3* (*NOS3*), and* KDR* (VEGF Receptor 2) genes were equally expressed in hPPCs and FMhMSCs, suggesting potential vasculogenic properties (Figures 3(d)–3(f)).

### 3.4. Vasculogenic Potential Assay

**Figure 4:**
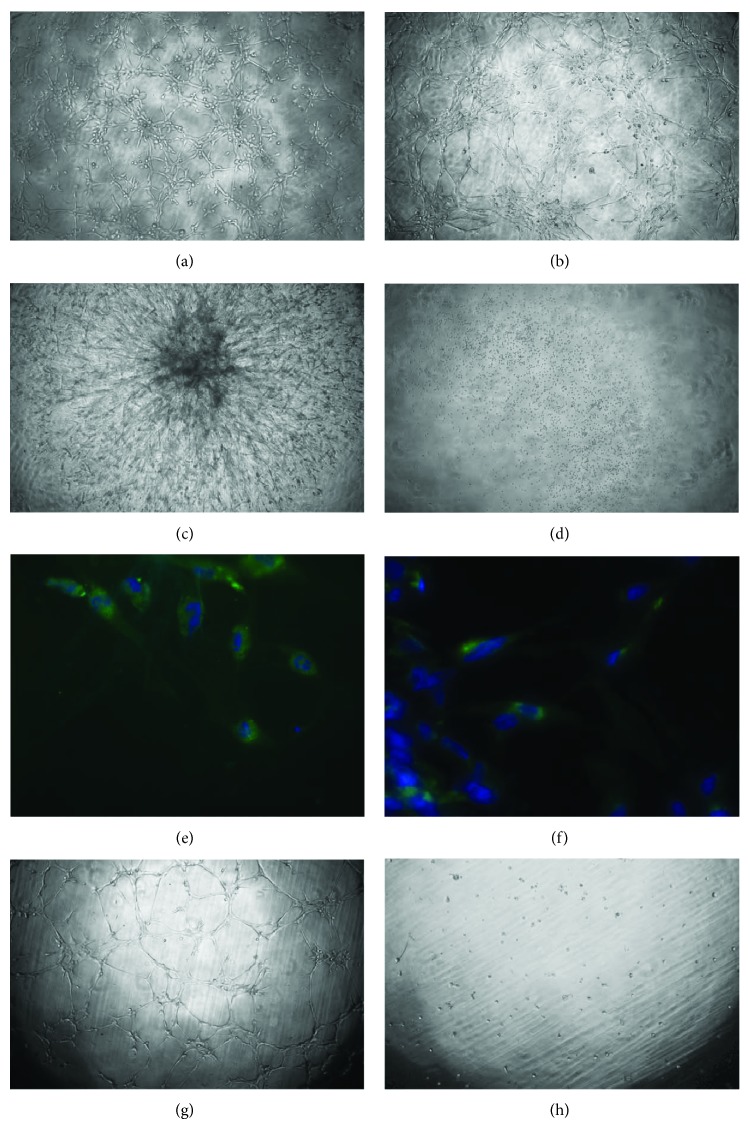
Vasculogenic potential of human Progenitor Perivascular Cells (hPPCs) assessment* in vitro*. (a)–(c) Capillary network formation in Matrigel (40x magnification) after 3 hours (a), 8 hours (b), and 7 days (c). (d) Assessment of vasculogenic potential in nonadherent cells with hematopoietic profile after 8 hours (40x magnification). (e)-(f) Immunofluorescence staining of cells involved in tubule-like structures recovered from Matrigel at 7 days: CD34-positive cells (*green*, (e)) and KDR-positive cells (*green*, (f)). Nuclei are counterstained with DAPI (*blue*). 400x magnification. (g)-(h) Assessment of vasculogenic potential in sorted cells: CD146+ (g) and CD146− cells (h) after 8 hours (40x magnification).

While we observed the ability of pericyte/MSC-like cells (Figures 4(a)–4(c)) to form capillary-like structures* in vitro*, occurring as early as within 3 hours (Figure 4(a)), no vasculogenic properties were observed in nonadherent cells with hematopoietic profile (Figure 4(d)).

Forty-eight hours after, the capillary network of pericyte/MSC-like cells collapsed and formed “sphere”-like structures that gradually spread out forming numerous capillaries, covering the surface of well after 1 week (Figure 4(c)). Immunofluorescence analysis performed one week after seeding in semisolid medium revealed the presence of CD34-positive (Figure 4(e)) and KDR-positive (Figure 4(f)) cells, indicating that hPPCs within the network-like structures were partially committed towards an endothelial phenotype.

Interestingly, sorted CD146+ cells showed the ability to form capillary-like structures* in vitro*, contrarily to CD146− cells that did not show vasculogenic properties (Figures 4(g)-4(h)).

**Figure 5:**
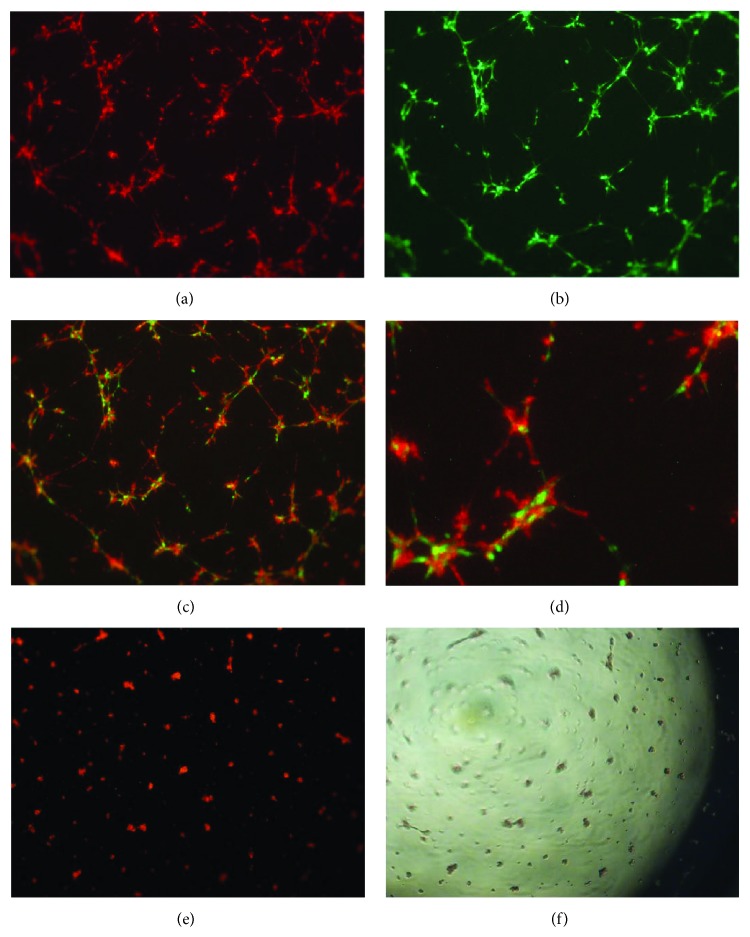
*In vitro* coculture of human Progenitor Perivascular Cells (hPPCs) and endothelial cells on Matrigel. (a)–(d) When cocultured with HUVECs (a), hPPCs (b) are able to exert the role of pericytes by incorporating in network-like structures and aligning with endothelial cells, as shown in merged images ((c) and (d) in greater detail); images are representative of 3 experiments of the 24-hour coculture. Before seeding, cells were labeled with fluorescent vital dyes, green PKH2 (hPPCs) and red PKH26 (HUVECs). (e)-(f) Culture of HUVECs alone results in degenerated network-like structures within 24 hours as shown in (e) (red fluorescence) and (f) (phase contrast). 40x magnification.

We next investigated whether hPPCs may exert their role of pericytes by coculturing them with HUVECs. In the presence of hPPCs, HUVECs increased both the stability of capillary-like tubes in semisolid medium and their organizational efficiency (Figure 5). Within the tubular network, most of the HUVECs were closely associated with hPPCs (Figures 5(c) and 5(d)), a feature resembling that described for hMSCs behaving as pericyte/nurse-like elements.

## 4. Discussion

Compelling evidence indicates that virtually all blood vessels (arterial and venous) in the body harbor perivascular cellular elements exhibiting* in vitro* a panel of cell surface markers overlapping those detected in isolated MSCs [29, 30]. Their ability to differentiate into major mesodermal cell lineages, together with their contribution in organ development and in vascular remodeling, prompted the use of perivascular stem/progenitor cells in postinjury regeneration [30].

Although the presence of circulating cells with pericyte or MSC traits has been reported in fetal or adult human blood [31, 32], the isolation of pericytes or pericyte precursors in adult peripheral blood remains under debate. Here, we show that human PBMCs encompass a population of CD146+/CD45+/CD31+ spindle-shaped elements, a pattern consistent with that expressed in early EPCs [19]. These cells can be selected and expanded* in vitro* from the original PBMCs culture in the presence of fibronectin and Mesenchymal Stem Cell medium. Interestingly, over time in culture the observed early EPC-like population acquired a fibroblastoid trait similar to the MSC morphology, according to previous observation of mesodermal marker vimentin presence in CFUs-Hill [33]. Further refinement of the phenotypic characteristics of the early EPC-like population revealed the presence of a consistent percentage of CD146+/PDGFRbeta+/NG2+ cells, typical for a pericytic pattern [30]. Interestingly, the weekly assessment of this phenotype revealed that the percentage of pericyte markers increased starting from PBMCs throughout 21 days, rising up to 16.6%.

Flow cytometry analyses, in expanded spindle-shaped cells, revealed that this predominant population was associated with the appearance of expression patterns for cell surface markers that unambiguously distinguish cells with both MSC and pericyte identity. In particular, the observed pattern CD105+, CD73+, CD44+, CD166+, CD90+, CD29+, CD146+, and PDGFRbeta+, in the absence of CD14, CD45, and CD34 expression, was superimposable to that pattern extensively detected in MSCs isolated from multiple human sources, including the bone marrow [26, 34], the adipose tissue [35, 36], the term placenta [22, 37, 38], and the amniotic fluid [39–41]. The currently investigated hPPCs also expressed a basal transcriptional profile of both stemness-related (*OCT-4*,* KLF-4*, and* NANOG*) and vasculogenic (*KDR* and* MMP-2*) genes, comparable with the expression detected in adipose- or placental-derived MSCs. This observation suggests a high degree of pluripotency and the potential for complex lineage commitment. Accordingly, the MSC-like population exhibited remarkable vasculogenic properties* in vitro*, as shown by the early generation and long-lasting persistence of a dense capillary-like network and by the presence of CD34 and KDR positive cells indicating the ability of hPPCs to be committed along an endothelial fate.

Moreover, within the overall MSC population, a subset of homogeneous CD146 positive cells could also be sorted. When functionally tested* in vitro*, these cells fully retained the ability to form vascular-like structures. In contrast, the vasculogenic potential was completely lacking in the MSC subfraction devoid of the CD146+ counterpart. This finding indicates that the pericytic component within MSCs was (i) functionally competent and (ii) necessary and sufficient to support the vasculogenic properties of the unsorted MSC population. Consonant with these considerations, coculture experiments revealed the ability of hPPCs to prolong the persistence of the capillary-like network provided by HUVECs.

So far, pericytes have been considered to be mainly vascular wall-resident cells [5, 29, 42]. Circulating pericytes have been only detected in a few studies [10, 43] without subsequent isolation and expansion* in vitro*. Moreover a significant increase in circulating pericytes has only been detected in cancer patients, compared with healthy control, and it has been used as a prognostic oncogenic marker without any chance for further deployment in a regenerative medicine context. Here, according to other studies we failed to detect traceable amounts of circulating pericyte-like elements. Nevertheless, we succeeded in obtaining functional pericytes* in vitro* from a circulating subset of PBMCs. Studies are on the way to investigate whether the vasculogenic properties of these pericytes observed* in vitro* may be retained* in vivo* in animal models of acute myocardial infarction and chronic hind limb ischemia. In the affirmative, the current data may pave the way to novel approaches in cardiovascular cell therapy.

## Figures and Tables

**Table 1:** Comparative cytofluorimetric analysis in hPPCs, FMhMSCs, and hASCs at passage 4.

Antigen	hPPCs	FMhMSCs	hASCs
CD45	1.1 ± 0.3	0.3 ± 0.1	0.6 ± 0.1
CD14	0.7 ± 0.5	0.8 ± 0.3	0.4 ± 0.2
CD34	1.1 ± 0.9	0.4 ± 0.2	0.7 ± 0.3
CD105	94.6 ± 1.3	95.1 ± 0.7	84.2 ± 4.7
CD73	96.4 ± 1.7	99.7 ± 0.2	99.9 ± 0.0
CD44	85.8 ± 5.5	97.8 ± 0.8	94.6 ± 1.3
CD166	77.6 ± 4.9	95.2 ± 2.2	99.8 ± 0.1
CD90	99.3 ± 0.4	84 ± 6.9	99.9 ± 0.1
CD29	99.6 ± 0.2	99.8 ± 0.1	99.8 ± 0.1
CD146	46.6 ± 11.5	81 ± 5.0	55.7 ± 12.8
PDGFRbeta	44.3 ± 9.9	73 ± 6.2	95.9 ± 2.3
